# Emission Profiles of Volatiles during 3D Printing with ABS, ASA, Nylon, and PETG Polymer Filaments

**DOI:** 10.3390/molecules27123814

**Published:** 2022-06-14

**Authors:** Wojciech Wojnowski, Mariusz Marć, Kaja Kalinowska, Paulina Kosmela, Bożena Zabiegała

**Affiliations:** 1 Department of Analytical Chemistry, Faculty of Chemistry, Gdańsk University of Technology, 80-233 Gdańsk, Poland; mariusz.marc@pg.edu.pl (M.M.); kaja.kalinowska@pg.edu.pl (K.K.); bozena.zabiegala@pg.edu.pl (B.Z.); 2 Department of Chemistry, Faculty of Mathematics and Natural Sciences, University of Oslo, 0371 Oslo, Norway; 3 Department of Polymer Technology, Faculty of Chemistry, Gdańsk University of Technology, 80-233 Gdańsk, Poland; paulina.kosmela@pg.edu.pl

**Keywords:** 3D printing, volatile organic compounds, VOCs, emissions, indoor air, thermoplastics

## Abstract

In this short communication we characterize the emission of volatile organic compounds (VOCs) from fused filament fabrication (FFF) 3D printing using four polymer materials, namely polyethylene terephthalate glycol-modified (PETG), acrylonitrile styrene acrylate (ASA), Nylon, and acrylonitrile butadiene styrene (ABS). Detailed emission profiles are obtained during thermal degradation of the polymers as a function of temperature and also in real-time during 3D printing. Direct quantitative measurement was performed using proton transfer reaction time-of-flight mass spectrometry (PTR-ToF-MS). Qualitative determination of the volatiles emitted from the printed elements at various temperatures was accomplished using gas chromatography-mass spectrometry (GC-MS). The emission rates of VOCs differ significantly between the different polymer filaments, with the emission from Nylon and PETG more than an order of magnitude lower than that of ABS.

## 1. Introduction

The widespread availability of consumer-grade fused filament fabrication (FFF) 3D printers has prompted investigations of the user’s exposure to emissions resulting from the operation of these devices. These emissions can include aerosol and volatile organic compounds (VOCs). Initially, researchers focused on assessing the former, establishing the real-time emission rates of particulates during 3D printing [1,2]. In recent years, however, there has been an increased interest in assessing the corresponding emission of volatiles [3,4] and its potential effects, including investigations of in vivo toxicity (to rats) [5].

When determining the emission of VOCs during printing using the established approach of sampling on sorptive material and off-line analysis using gas chromatography-based methods, a major limitation is the low temporal resolution of the emission profiles. Certainly, it cannot match the direct measurements of aerosol concentration. This is why we have previously proposed an approach in which the volatiles emitted during FFF 3D printing are determined directly, using proton transfer reaction time-of-flight mass spectrometry (PTR-ToF-MS) [6]. This allows establishing real-time emission profiles of a multitude of volatile organic compounds both as a function of temperature during thermal degradation of the polymer filament and, perhaps more importantly, during actual 3D printing. The correct identification of the compounds is assured by performing a complementary qualitative analysis using gas chromatography-mass spectrometry (GC-MS). We have demonstrated this approach by assessing the emissions from 3D printing with the most ubiquitous FFF material, i.e., polylactide (PLA).

In this short communication, we investigate the VOCs emission profiles of four different FFF materials during 3D printing: polyethylene terephthalate glycol-modified (PETG), acrylonitrile styrene acrylate (ASA), Nylon, and acrylonitrile butadiene styrene (ABS). Additionally, a qualitative analysis of the emission of volatiles from the printed objects at four different temperatures was performed, together with thermogravimetric analysis.

## 2. Results and Discussion

**Figure 1:**
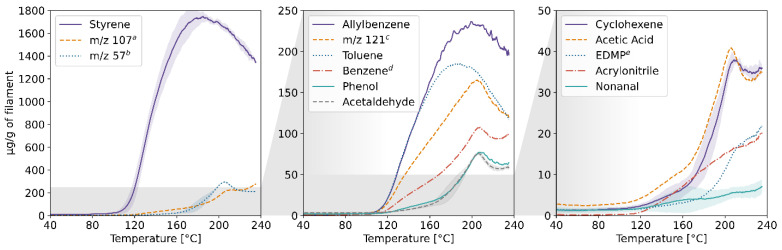
Temperature-dependent emission profile of the ABS filament. The grey horizontal field indicates the area magnified in the following sub-plot, and the coloured shaded areas indicate SD (*n* = 3, only selected ones shown for clarity). A rolling average of *n* = 10 data points. ^a^ *m/z* 107: benzaldehyde, C_8_-alkylbenzenes; k = 2.24 × 10^−9^ cm^3^s^−1^; ^b^ *m/z* 57: product of butanol fragmentation in the PTR-MS drift chamber; ^c^ *m/z* 121: acetophenone, C_9_-alkylbenzenes; k = 2.40 × 10^−9^ cm^3^s^−1^; ^d^ likely a product of C_9_-alkylbenzenes fragmentation in the PTR-MS drift chamber; ^e^ 3-ethyl-2,5-dimethyl-pyrazine.

**Figure 2:**
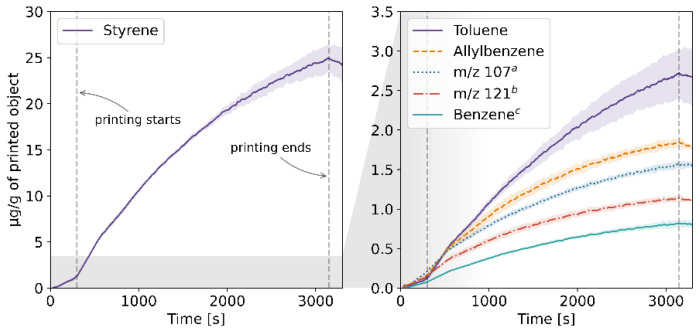
The mixing ratio of main VOCs within the enclosure during FFF 3D printing with the ABS filament. ^a,b,c^ see Figure 1 caption.

**Figure 3:**
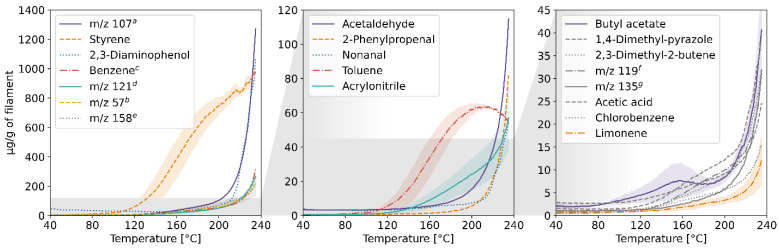
Temperature-dependent emission profile of the ASA filament. The legend in subplot 3 is arranged from highest to lowest emission at 240 °C. ^a,b,c^ see Figure 1 caption; ^d^ acetophenone, C_9_-alkylbenzenes; k = 2.40 × 10^−9^ cm^3^s^−1^; ^e^ unidentified; k = 2.0 × 10^−9^ cm^3^s^−1^; ^f^ α-methylstyrene, phenylpropene; k = 2.0 × 10^−9^ cm^3^s^−1^; ^g^ tert-butyllbenzene, p-propyltoluene; k = 2.0 × 10^−9^ cm^3^s^−1^.

**Figure 4:**
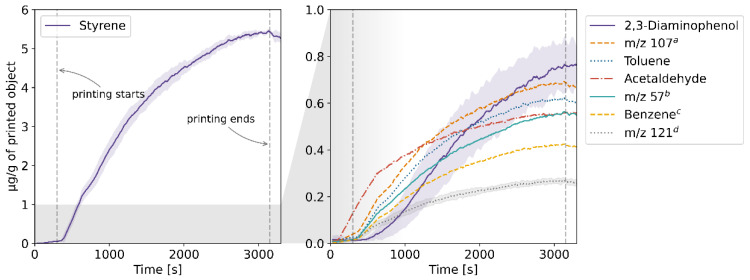
The mixing ratio of main VOCs within the enclosure during FFF 3D printing with the ASA filament. ^a,b,c,d^ see Figure 3 caption.

**Figure 5:**
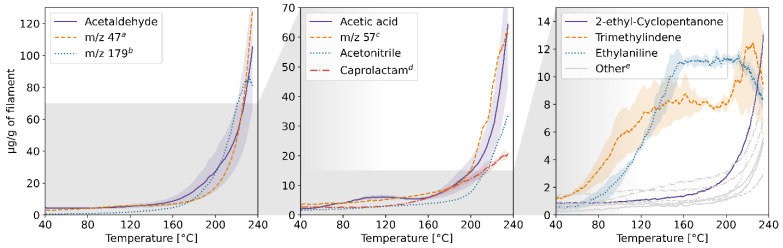
Temperature-dependent emission profile of the Nylon filament. ^a^ unidentified, possibly ethanol; ^b^ unidentified; ^c^ *m/z* 57: product of butanol fragmentation in the PTR-MS drift chamber; ^d^ not identified in the TD-GC-MS spectra; ^e^ see Table S1.

**Figure 6:**
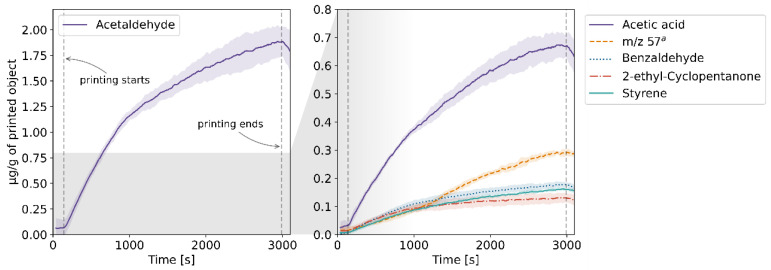
The mixing ratio of main VOCs within the enclosure during FFF 3D printing with the Nylon filament. ^a^ see Figure 5 caption.

**Figure 7:**
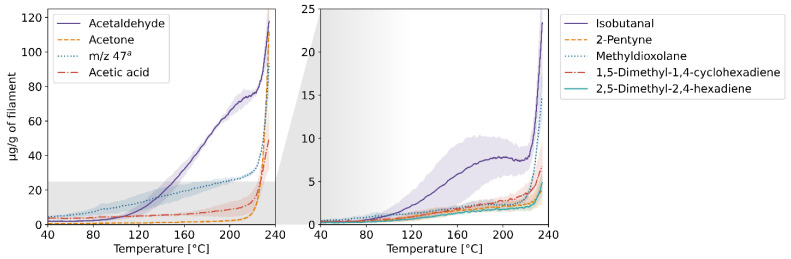
Temperature-dependent emission profile of the PETG filament. ^a^ unidentified, possibly formic acid; k = 1.99 × 10^−9^ cm^3^s^−1^.

**Figure 8:**
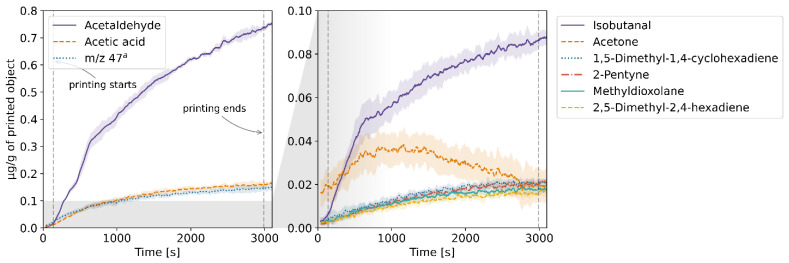
The mixing ratio of main VOCs within the enclosure during FFF 3D printing with the PETG filament. ^a^ see Figure 7 caption.

The results of the TD-GC-MS qualitative analysis were juxtaposed with the PTR-MS mass spectra of real-time measurements. The compounds which were identified and subsequently monitored using PTR-MS are listed in Table S1 in the Supplementary Materials. The table also contains information on whether the compound was identified in samples collected at a given temperature. The emission rates of these volatiles in relation to the temperature for each of the four filaments and the corresponding profiles of emission during 3D printing are plotted in Figure 1, Figure 2, Figure 3, Figure 4, Figure 5, Figure 6, Figure 7 and Figure 8. Note that some plots show the tentative emission profile of compounds that, due to the indirect sampling, were not identified using TD-GC-MS, e.g., acetaldehyde. Furthermore, in cases in which it was difficult to discriminate between isobaric and isomeric compounds (e.g., ethylbenzene, xylenes, benzaldehyde), the tentative signal for the corresponding *m/z* is plotted instead. The signals corresponding to some ions which were not identified based on the TD-GC-MS analysis and the monoisotopic mass, but which were nonetheless prominent in the PTR-MS spectra (e.g., *m/z* 158 in Figure 3), were also plotted.

It should be noted that this temperature-related emission characteristic was obtained through dynamic headspace sampling, in conditions more closely resembling the corresponding TG analysis, then real-life application in FFF 3D printing. In this regard, the results of measurements carried out during actual printing, shown in Figure 2, Figure 4, Figure 6 and Figure 8, are a better indicator of the potential user’s exposure to the identified VOCs.

In the case of ABS, styrene (classified by the International Agency for Research on Cancer (IARC) as “probably carcinogenic to humans”—Group 2A) is the main VOC emitted during printing, which is in line with previously reported results, including our findings [7,8,9]. Benzene (classified by IARC as “carcinogenic to humans”—Group 1) was detected in the PTR-MS mass spectra, but not in the results of TD-GC-MS analysis which suggests that it is predominantly a product of fragmentation of alkylbenzenes (ethylbenzene, xylenes) during ionization in the PTR-MS. However, it was previously reported as one of the VOCs emitted during 3D printing using ABS [10]. The emission rate of styrene alone during the dynamic sampling measurement reached nearly 1.8% of the total mass of the sample.

The composition of the emission from the ASA filament is not dissimilar from that of ABS. However, unlike with ABS, the emission rate of styrene and some other VOCs does not peak at approx. 200 °C. Styrene remained the predominant volatile compound emitted during printing; however, its emission rate was less than a quarter the emission of styrene from ABS. While all the tested filaments did not contain colourants, they are by no means comprised solely of their eponymous polymers, as evidenced by the emission of D-limonene which can be used as a solvent during the manufacture of plastics [11]. In the case of both ABS and ASA, the background emission of acetaldehyde and acetic acid from the polylactide elements of the printer was high enough compared to the overall emission observed during printing to hinder reliable estimation of the mixing ratio of these compounds. The background emission was determined during a blank measurement, i.e., printing without filament in the extruder.

In the case of Nylon, the overall emission rates during dynamic sampling (see Figure 5) were an order of magnitude lower than in the case of ABS and ASA, and fewer compounds were identified in the sample’s volatile fraction. The *m/z* 47 ion in Figure 5 might be indicative of the emission of ethanol, in which case its mixing ratio was likely underestimated due to fragmentation during ionization in the PTR-MS drift chamber [12]. Davis et al. and Azimi et al. previously reported that the main VOC emitted during FFF printing using Nylon filaments is caprolactam [10,13]; however, we were not able to corroborate that in this study.

The emission from the PETG filament was the lowest among the four tested filaments both in terms of the overall rate and the number of identified compounds. The main emitted VOC was acetaldehyde. Gu et al. [14] and Floyd et al. [15] reported the main VOCs emitted during printing using PETG to be acetic acid and D-limonene, respectively. The reason for this discrepancy might be that acetaldehyde is difficult to sample using Tenax TA which hinders TD-GC-MS determination of this compound, as was the also case in this study. While the background emission of acetone from the elements of the 3D printer was accounted for by subtracting a blank measurement (without the filament), it might still have affected the emission profile of this compound shown in Figure 8.

**Figure 9:**
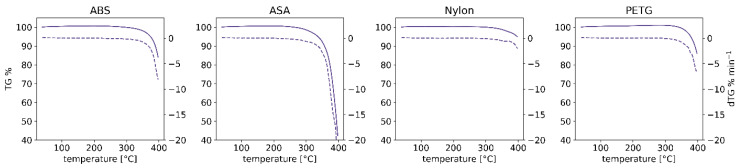
Results of the thermogravimetric analysis of the four FFF filaments.

The mass of the samples did not change significantly in the monitored temperature range (40 °C–240 °C) during TG analysis (see Figure 9), which suggests that the emission of volatiles monitored during the dynamic sampling experiments represents a large fraction of the overall emission. However, due to mechanical factors, this might not necessarily be the case during FFF extrusion, in which case the emission of particulates and SVOCs (semi-volatile organic compounds) might play a greater role [16]. 

## 3. Materials and Methods

### 3.1. Samples

Samples of 1.75 mm FFF 3D-printing filaments were obtained from online vendors in Poland. All were marketed as “natural”, i.e., without added colourants. They included: polyethylene terephthalate glycol-modified (PETG) (Print-Me, Gorzów Wielkopolski, Poland), acrylonitrile styrene acrylate (ASA) (Print-Me, Gorzów Wielkopolski, Poland), Nylon (Print-Me, Gorzów Wielkopolski, Poland), and acrylonitrile butadiene styrene (ABS) (Nebula Filaments, Stare Bystre, Poland). The samples were stored at room temperature in factory-sealed airtight packaging prior to the experiments. The printed objects (rectangular cuboids) were wrapped in aluminium foil and stored at −20 °C prior to TD-GC-MS analysis.

### 3.2. Estimation of the Emission Rates of VOCs from the Various Filaments in Relation to Temperature and during FFF 3D-Printing Using PTR-ToF-MS

A detailed description of the experimental setup used to assess in real-time the emission profile of a gradually heated filament fragment and also during 3D printing can be found in previous work [6]. Briefly, in order to establish the emission profile of a particular polymer filament as a function of temperature, a small segment of the filament (approx. 5 mm, 15 mg) was gradually heated in a 20 mL sealed glass headspace vial to 240 °C, with the sample headspace dynamically drawn at 50 mL∙min^−1^ into the transfer line of the PTR-ToF-MS and afterward diluted 20× with zero air (approx. 5.5 N). The measurement was conducted in real-time, with the mass spectra integrated every second. Unlike the previously described setup, the zero air was supplied from a zero air generator. 

The same filament was then loaded into an FFF 3D printer (Prusa i3 MK2S, Prusa Research a.s., Prague, Czech Republic) which was placed in an airtight 130 dm^3^ enclosure. The 3D printer was programmed to print a 30 × 30 × 20 mm cuboid, with a layer height of 0.2 mm, the wall line width of 0.4 mm (the nozzle diameter), the number of wall lines and top and bottom layers set to 2, and an infill of 20%. The print speed was 40%, and the fan which cools the deposited polymer was run at 20% speed when printing with Nylon, 100% when printing with PETG, and 30% when printing with ASA and ABS, following the manufacturer’s guidelines. The print platform was heated to 80 °C, and the nozzle was heated to 240 °C when printing with ASA, ABS, and PETG, and to 250 °C when printing with Nylon, again following the manufacturer’s recommendation. The air within the enclosure was sampled into the PTR-ToF-MS through a heated transfer line at 100 mL∙min^−1^ and without dilution. Again, the measurement was conducted in real-time, and the spectra were integrated every 3 s.

In both cases, the mixing ratios of the emitted volatile organic compounds were estimated using the PTR TOF 1000 Ultra (Ionicon Analytik GmbH, Innsbruck, Austria) PTR-ToF-MS. The temperature of the transfer line and the drift chamber was set to 70 °C, the drift chamber voltage and pressure were maintained at 610 V and 2.60 mbar, respectively, and the E/N was at 120 Td (1 Td = 10^−21^ V m^–2^). The compounds were identified primarily based on the results of the TD-GC-MS analysis, but also on the exact *m/z* of the monitored ions and the isotopic ratios. A threshold of 5 cps was used. The mixing ratios of the monitored ions were estimated based on the reaction kinetics with the hydronium ions, with the literature values for the proton transfer reaction rate k [17,18,19,20] assigned to the identified ions. Unidentified ions were assigned k = 2.0 × 10^−9^ cm^3^s^−1^. This approach typically entails an uncertainty of approx. 25% [21]. The mass spectra were processed using the PTR-MS Viewer version 3.4.3.12 software (Ionicon Analytik GmbH, Innsbruck, Austria). 

### 3.3. TD-GC-MS Sampling and Qualitative Analysis

A screening study of the emission of VOCs from the 3D-printed polymer cuboids was performed using the Micro-Chamber/Thermal Extractor™ (µ-CTE™ 250, Markes International Ltd., Bridgend, UK) system. Detailed information about the working parameters of the setup was specified elsewhere [22,23,24]. The 3D-printed samples were placed inside the chamber and conditioned at four temperatures: 40 °C, 80 °C, 120 °C, and 160 °C. Emitted compounds were collected for 5 min on a Tenax TA sorption material under a constant flow of nitrogen at a rate of 25 ± 1 mL∙min^−1^. The analytes were desorbed from Tenax TA using thermal desorption (TD Unity v.2, Markes International Ltd., Llandrisant, UK) under the following conditions: (i) the sorption tube was heated up to 290 °C (±3 °C) and held at that temperature for 15 min under the inert gas flow (helium, 50 mL∙min^−1^)—analytes were transferred directly to the multibed microtrap (1 °C); (ii) next, the microtrap was rapidly heated to 300 °C for 5 min and the analytes were transferred directly to the GC capillary column (J&W, HP-1MS 30 m × 0.25 mm × 1 µm, Agilent Technologies, Santa Clara, CA, USA) in a stream of inert gas (helium at a flow rate of 1.0 mL∙min^−1^). The analysis was performed using a gas chromatograph (Agilent Technologies 6890) combined with a mass spectrometer (5873 Network Mass Selective Detector, Agilent Technologies). The compounds were identified by comparing the spectra with the NIST Mass Spectral Library v. 2.0 (2011). Working parameters of the GC-MS system were as follows: oven program: initial temperature: 50 °C maintained for 1 min, next ramped at a rate of 10 °C∙min^−1^ to 120 °C and held for 2 min, and finally ramped at a rate of 15 °C·min^−1^ to 260 °C and maintained for 5 min; MS ion source temperature: 230 °C; quadrupole mass analyser temperature: 150 °C; GC-MS transfer line temperature: 280 °C, scan mode—total ion chromatogram (from 35 to 500 *m/z*). 

A sketch depicting the experimental setup is shown in Figure S1 in the Supplementary Materials.

### 3.4. Thermogravimetric Analysis

The thermogravimetric analysis (TGA) of the polymer filaments was performed using the TG 209 F3 instrument (Netzsch, Selb, Germany). Filament samples weighing approx. 10 mg were placed in a ceramic dish. The analysis was performed in synthetic air (21% and 79% of pure oxygen and nitrogen, respectively) with the temperature ramped from 35 °C to 400 °C at the rate of 10 °C∙min^−1^.

## 4. Conclusions

The chosen approach for the qualitative and quantitative determination of the emission of VOCs during 3D printing and thermal degradation of the polymer filaments produced good and repeatable results. Of the four tested FFF 3D printing materials, by far the highest emissions were observed when printing with ABS, in which case the emission profile was dominated by the main VOC, styrene (up to 25 µg·g^−1^ of the printed object). The overall emission when printing with Nylon and PETG was more than an order of magnitude lower than in the case of ABS. Since printed elements made of both Nylon and PETG have mechanical properties similar to those of ABS [25], they should be strongly considered as alternatives in consumer-level use of FFF, where proper ventilation cannot always be assured.

## Data Availability

The data presented in this study are available in the Supplementary Materials and raw data are available on request from the corresponding author.

## Supplementary Materials

The following supporting information can be downloaded at: https://www.mdpi.com/article/10.3390/molecules27123814/s1, Figure S1: Sampling setup for the real-time monitoring of the emission of VOCs during 3D printing (a), establishing the temperature-dependant emission profile of FFF 3D printing filaments (b), and for identification of VOCs emitted from 3D printed objects at different temperatures (c, d–thermal extractor/micro-chamber); 1. PTR-ToFMS; 2. heated transfer line; 3. ventilated enclosure housing an FFF 3D printer; 4. thermostated block with a headspace vial containing a filament fragment, coupled with the PTR-ToF-MS via the transfer line; 5. empty reference vial with a thermocouple; 6. laboratory heater; 7. Tenax TA sorption tube; 8. 3D printed object; Table S1: Compounds emitted from ABS, ASA, Nylon, and PETG cuboids identified at different temperatures in both the TD-GC-MS and PTR-ToF-MS spectra. The PTR-MS *m*/*z* column denotes the calculated value.

## Sample Availability

Samples of the compounds are not available from the authors.
