# Supplementary material for: Emission Profiles of Volatiles during 3D Printing with ABS, ASA, Nylon, and PETG Polymer Filaments
Source: Molecules. 2022 Jun 14;27(12):3814. doi: 10.3390/molecules27123814 (PMC9229569; doi:10.3390/molecules27123814)
Supplement: Supplementary file 1 [file molecules-27-03814-s001.zip › molecules-1763099-supplementary.pdf]

Supplemetary Information for

Emission profiles of volatiles during 3D printing with ABS, ASA, Nylon, and PETG polymer filaments

Wojciech Wojnowski [1,2,*], Mariusz Marć [1], Kaja Kalinowska [1], Paulina Kosmela [3], and Bożena Zabiegała [1]

1 Department of Analytical Chemistry, Faculty of Chemistry, Gdańsk University of Technology, Gdańsk, Poland;
2 Department of Chemistry, Faculty of Mathematics and Natural Sciences, University of Oslo, Oslo, Norway;
3 Department of Polymer Technology, Faculty of Chemistry, Gdańsk University of Technology, Gdańsk, Poland.
* Correspondence: wojciech.wojnowski@pg.edu.pl

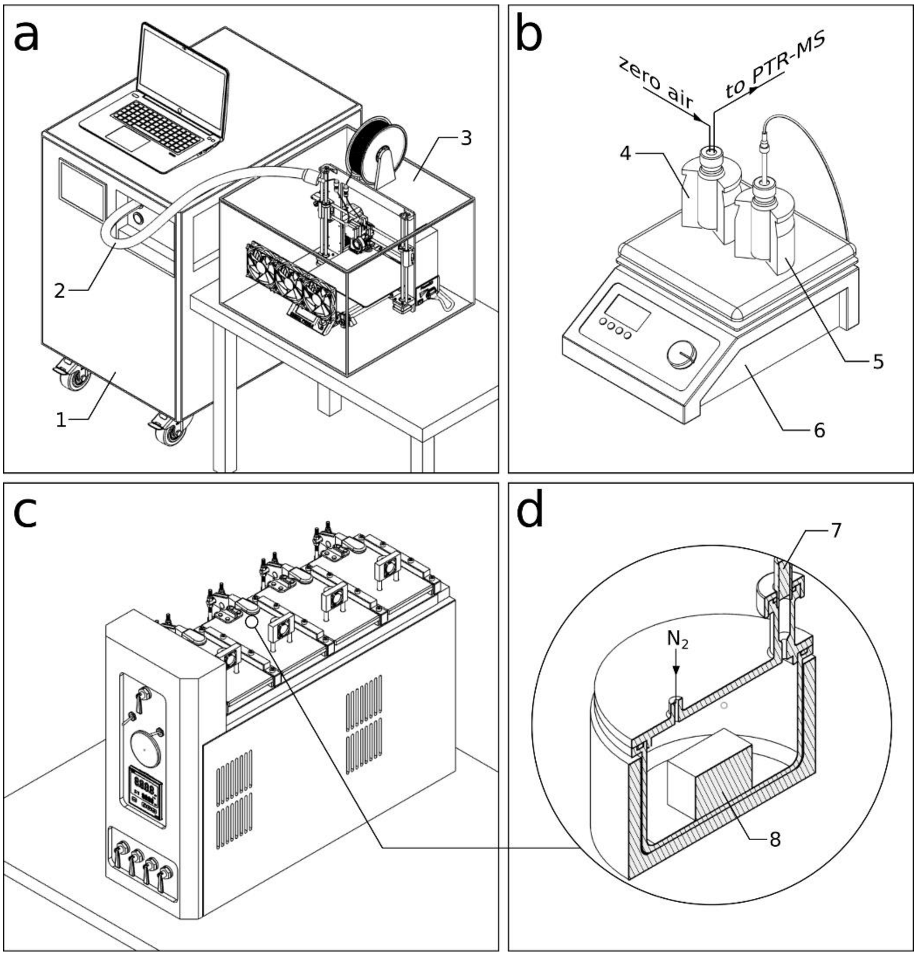

Figure S1. Sampling setup for the real-time monitoring of the emission of VOCs during 3D printing (a), establishing the temperature-dependant emission profile of FFF 3D printing filaments (b), and for identification of VOCs emitted from 3D printed objects at different temperatures (c, d – thermal extractor/micro-chamber) ; 1. PTR-ToF-MS; 2. heated transfer line; 3. ventilated enclosure housing an FFF 3D printer; 4. thermostated block with a headspace vial containing a filament fragment, coupled with the PTR-ToF-MS via the transfer line; 5. empty reference vial with a thermocouple; 6. laboratory heater; 7. Tenax TA sorption tube; 8. 3D printed object.

Table S1. Compounds emitted from ABS, ASA, Nylon, and PETG cuboids identified at different temperatures in both the TD-GC-MS and PTR-ToF-MS spectra. The PTR-MS m/z column denotes the calculated value.

Name	formula	PTR-MS m/z	RT	TD-GC-MS			
				40°C	80°C	120°C	160°C
**ABS**							
Acrylonitrile	C_3H_3N	54.034	2.77	✓	✓	✓	✓
Acetic acid	$C_2H_4O_2$	61.028	3.28	✓	✓	✓	✓
Butanol	$C_4H_{10}O$	75.080	4.21	✓	✓	✓	✓
Cyclohexene	C_6H_{10}	83.085	7.34			✓	✓
Toluene	C_7H_8	93.070	6.03		✓	✓	✓
Phenol	C_6H_6O	95.049	17.5	✓	✓	✓	✓
Styrene	C_8H_8	105.070	8.21	✓	✓	✓	✓
Benzaldehyde	C_7H_6O	107.049	9.42			✓	✓
Ethylbenzene	C_8H_{10}	107.085	7.73			✓	✓
p,m-Xylene	C_8H_{10}	107.085	8.22			✓	✓
α-Methylstyrene	C_9H_{10}	119.085	10.24				✓
Phenylpropene	C_9H_{10}	119.085	11.23				✓
Acetophenone	C_8H_8O	121.065	11.78			✓	✓
Cumene	C_9H_{12}	121.101	9.02			✓	✓
Propylbenzene	C_9H_{12}	121.101	9.65			✓	✓
3-Ethyl-2,5-dimethyl-pyrazine	$C_8H_{12}N_2$	137.107	12.04			✓	✓
Nonanal	$C_9H_{18}O$	143.143	12.44	✓	✓	✓	✓
**ASA**							
Acrylonitrile	C_3H_3N	54.034	2.77	✓	✓	✓	✓
Acetic acid	$C_2H_4O_2$	61.028	3.28	✓			✓
Butanol	$C_4H_{10}O$	75.080	4.21	✓	✓	✓	✓
2,3-Dimethyl-2-butene	C_6H_{12}	85.101	3.30	✓	✓	✓	✓
Toluene	C_7H_8	93.070	6.03	✓	✓	✓	✓
1,4-Dimethyl-pyrazole	$C_5H_8N_2$	97.076	6.79			✓	✓
Styrene	C_8H_8	105.070	8.21	✓	✓	✓	✓
Benzaldehyde	C_7H_6O	107.049	9.42	✓	✓	✓	✓
Ethylbenzene	C_8H_{10}	107.085	7.73	✓	✓	✓	✓
p,m - Xylene	C_8H_{10}	107.085	8.22		✓	✓	✓
Chlorobenzene	C_6H_5Cl	113.015	7.4			✓	✓
Butyl acetate	$C_6H_{12}O_2$	117.091	6.62			✓	✓
α-Methylstyrene	C_9H_{10}	119.085	10.24			✓	✓
Phenylpropene	C_9H_{10}	119.085	10.44		✓	✓	✓
Acetophenone	C_8H_8O	121.065	11.78		✓	✓	✓
Cumene	C_9H_{12}	121.101	9.02				✓
1,2,4-Trimethylbenzene	C_9H_{12}	121.101	10.00				✓
Propyl benzene	C_9H_{12}	121.101	9.65	✓	✓	✓	✓
1-Ethyl-3-methyl-benzene	C_9H_{12}	121.101	9.83			✓	✓
1-Ethyl-2-methyl-benzene	C_9H_{12}	121.101	9.88				✓
2-phenylpropenal	C_9H_8O	133.067	13.27				✓
tert-Butylbenzene	$C_{10}H_{14}$	135.117	10.58		✓	✓	✓
p-propyltoluene	$C_{10}H_{14}$	135.117	11.00		✓	✓	✓
D-Limonene	$C_{10}H_{16}$	137.132	11.49				✓
Nonanal	$C_9H_{18}O$	143.143	12.44		✓	✓	✓
**Nylon**							
Acetic acid	$C_2H_4O_2$	61.028	3.28	✓	✓	✓	✓
Butanol	$C_4H_{10}O$	75.080	4.21	✓	✓	✓	✓
Cyclopropene, 3-methyl-3-vinyl-	C_6H_8	81.070	2.87	✓	✓	✓	✓
2-Methyl-1,3-dioxolane	$C_4H_8O_2$	89.059	4.02			✓	✓

Name	formula	PTR-MS m/z	RT	TD-GC-MS 40°C	80°C	120°C	160°C
Styrene	C_8H_8	105.070	8.21	✓	✓	✓	✓
Benzaldehyde	C_7H_6O	107.049	9.42	✓	✓	✓	✓
2-Ethylcyclopentanone	$C_7H_{12}O$	113.096	11.21		✓	✓	✓
Ethylaniline	$C_8H_{11}N$	122.096	13.3			✓	✓
Octanal	$C_8H_{16}O$	129.127	10.44	✓	✓	✓	✓
Nonanal	$C_9H_{18}O$	143.143	12.48	✓	✓	✓	✓
Decanal	$C_{10}H_{20}O$	157.158	14.07	✓	✓	✓	✓
1,2,3-Trimethylindene	$C_{12}H_{14}$	159.117	16.66			✓	✓
**PETG**							
Acetic acid	$C_2H_4O_2$	61.028	3.28	✓	✓	✓	✓
2-Pentyne	C_5H_8	69.070	2.74			✓	✓
Isobutanal	C_4H_8O	73.065	3.01			✓	✓
Methyldioxolane	$C_4H_8O_2$	89.059	4.03		✓	✓	✓
1,5-Dimethyl-1,4-cyclohexadiene	C_8H_{12}	109.101	7.68	✓	✓	✓	✓
2,5-Dimethyl-2,4-hexadiene	C_8H_{14}	111.117	6.37		✓	✓	✓
